# StemSC: a cross-dataset human stemness index for single-cell samples

**DOI:** 10.1186/s13287-022-02803-5

**Published:** 2022-03-21

**Authors:** Hailong Zheng, Jiajing Xie, Kai Song, Jing Yang, Huiting Xiao, Jiashuai Zhang, Keru Li, Rongqiang Yuan, Yuting Zhao, Yunyan Gu, Wenyuan Zhao

**Affiliations:** 1 Department of Systems Biology, College of Bioinformatics Science and Technology, Harbin Medical University, Harbin, 150086 China; 2 Department of Bioinformatics, School of Basic Medical Sciences, Southern Medical University, Guangzhou, 510515 China; 3 National Institute for Data Science in Health and Medicine, Xiamen University, Xiamen, 361005 China

**Keywords:** Stemness, Single-cell analysis, Cross-dataset, Cell dedifferentiation, Tumor microenvironment

## Abstract

**Background:**

Stemness is defined as the potential of cells for self-renewal and differentiation. Many transcriptome-based methods for stemness evaluation have been proposed. However, all these methods showed low negative correlations with differentiation time and can’t leverage the existing experimentally validated stem cells to recognize the stem-like cells.

**Methods:**

Here, we constructed a stemness index for single-cell samples (StemSC) based on relative expression orderings (REO) of gene pairs. Firstly, we identified the stemness-related genes by selecting the genes significantly related to differentiation time. Then, we used 13 RNA-seq datasets from both the bulk and single-cell embryonic stem cell (ESC) samples to construct the reference REOs. Finally, the StemSC value of a given sample was calculated as the percentage of gene pairs with the same REOs as the ESC samples.

**Results:**

We validated the StemSC by its higher negative correlations with differentiation time in eight normal datasets and its higher positive correlations with tumor dedifferentiation in three colorectal cancer datasets and four glioma datasets. Besides, the robust of StemSC to batch effect enabled us to leverage the existing experimentally validated cancer stem cells to recognize the stem-like cells in other independent tumor datasets. And the recognized stem-like tumor cells had fewer interactions with anti-tumor immune cells. Further survival analysis showed the immunotherapy-treated patients with high stemness had worse survival than those with low stemness.

**Conclusions:**

StemSC is a better stemness index to calculate the stemness across datasets, which can help researchers explore the effect of stemness on other biological processes.

**Supplementary Information:**

The online version contains supplementary material available at 10.1186/s13287-022-02803-5.

## Background

Stemness is defined as the self-renewal and differentiation potential of cells [1]. Cancer progression is accompanied by the acquisition of this feature [2]. Besides, quantification of stemness is very helpful to reconstruct cellular differentiation trajectories and explore the role of stemness in tumor tissues [3]. As reported, by using the stemness index, pervasive negative associations were found between cancer stemness and anticancer immunity [4].

With the increasing number of RNA-seq data, many transcriptome-based methods for stemness evaluation have been proposed, such as mRNAsi [5]. However, this method was only trained from bulk data, which limited its performance in single-cell data [3]. Gunsagar S. Gulati has proposed a more suitable method for single cells, named CytoTRACE [3], which showed better performance than all the currently known methods. However, the average correlation between this stemness index and differentiation time was only about 0.6. What’s more, the xenotransplantation assay has become the gold standard assay to define cancer stem cells (CSCs) in tumor cells [6], which is laborious, costly and rare. Lack of the experimentally validated stem cells makes the transcriptome-based methods can’t recognize stem-like cells by comparison because these transcriptome-based methods were too vulnerable to batch effect [7] to make use of the existing CSC samples. Therefore, it is necessary to construct a stemness index that is highly correlated with differentiation time and could be evaluated for single-cell samples across datasets.

In our previous studies, we have identified many signatures based on relative expression orderings (REOs) of gene pairs [8–10], which were not sensitive to batch effects and can be robustly applied to independent validation sets. Based on these unique advantages, we constructed a REO-based stemness index based on bulk samples [7], which showed a high correlation with differentiation time. However, the lack of single-cell samples in its training sets limited its performance in the collected single-cell samples of this study.

In this study, we constructed a REO-based stemness index for single-cell samples, StemSC, which can be used across datasets. Then, we validated StemSC by its higher negative correlations with differentiation time and higher positive correlations with tumor dedifferentiation than CytoTRACE in eight independent datasets and the merged datasets. By using StemSC, we recognized the tissue-specific stem genes and constructed cell differentiation trajectories automatically. Especially for tumor cells, StemSC can leverage the existing transcriptome data of the experimentally validated CSCs to recognize the stem-like cells in other independent datasets. Finally, cell–cell communication analysis showed that the recognized stem-like cells had fewer connections with each other and the immune cells than the common tumor cells. Further survival analysis showed that, for the immunotherapy-treated patients, the high-stemness group had worse overall survival than the low-stemness group.

## Methods

### Data and preprocessing

In this study, we downloaded the gene expression data of 11 human embryonic stem cell (ESC) datasets to reveal the high stability of REOs (Additional file 1: Table S1). We also downloaded six datasets with differentiation time (Additional file 1: Table S2) for identifying stemness-related genes and 13 datasets (Additional file 1: Table S3) for the development of StemSC. Five independent datasets with differentiation time were downloaded to validate the performance of StemSC (Additional file 1: Table S4). Three colorectal cancer datasets and four glioma datasets were also downloaded to validate the performance of StemSC in tumor cells (Additional file 1: Table S4). We excluded the samples of distant metastatic tumor to focus on the corresponding cancer type. Especially for the glioma dataset GSE117891, we excluded the cells from the normal tissues because these cells were limited to few patients.

For the RNA-seq expression data of both the bulk and single-cell samples, we directly downloaded the processed RPKM, TPM or count data from the Gene Expression Omnibus [11] (GEO, https://www.ncbi.nlm.nih.gov/geo/), Sequence Read Archive (SRA, https://www.ncbi.nlm.nih.gov/sra/), and Progenitor Cell Biology Consortium [12] (PCBC, https://www.synapse.org/#!Synapse:syn1773109/wiki/54962). Count data were turned to the RPKM data with the corresponding reference genomes of the datasets. Due to the filtered genes in GSE57872, we downloaded raw single-cell RNA sequencing data from SRA accession SRP042161 [13]. To retrieve the transcriptomic profiles of GSE57872, we built a reference transcriptome based on the GENCODE v19 annotation [14] and mapped the paired-end 25 bp reads to the reference transcriptome by using HISAT2 [15] (version 2.1.0, with parameters -q -p 1 -5 0 -3 0 -k 5 –min-intronlen 20 –max-intronlen 500000 –phred33). The RPKM data of GSE57872 were calculated by using featureCount [16] (with parameters -t exon -g gene_id –primary). Then, for each RNA-seq expression dataset, we mapped the Ensembl gene ID or gene symbol to the Entrez gene ID by using the reference downloaded from HUGO Gene Nomenclature Committee (HGNC, https://www.genenames.org/download/custom/). Dataset PRJNA482620 was preprocessed in the same way. Especially, we removed the low-quality cells with less than 2000 detected genes for single-cell data.

### Consistency evaluation of REOs between datasets

In this study, we calculated the REOs by using the overlapping genes among the datasets with more than three samples. Pairwise comparisons were performed for the expression level of the above genes for each sample. For each gene pair (*G*_*i*_, *G*_*j*_), we retained the gene pair with certain REO (*G*_*i*_ > *G*_*j*_ or *G*_*i*_ < *G*_*j*_) in all samples of the dataset, which we called stable REO. The consistency of stable REOs between two datasets was calculated as *s*/*n*, where *n* was the number of shared gene pairs between the stable REOs of two datasets and *s* was the number of shared gene pairs with the same REOs. The significance of consistency was determined by the cumulative binomial distribution model as follows:

$$P=1-\sum_{i=0}^{s-1}\left(\genfrac{}{}{0pt}{}{n}{i}\right){\left({P}_{0}\right)}^{i}{\left(1-{P}_{0}\right)}^{n-i}$$

where *P*_0_ is the probability that gene pairs showed same REO pattern (*G*_*i*_ > *G*_*j*_ or *G*_*i*_ < *G*_*j*_) in two datasets by chance (*P*_0_ = 0.5).

### Development of the StemSC

The stemness index for single-cell data, StemSC, was constructed by calculating the similarity of REOs between target cells and ESC samples. Firstly, we identified the stemness-related genes by selecting the genes significantly related to differentiation time in all five datasets (Additional file 1: Table S2). Then, due to the lack of single-cell data, we used 13 RNA-seq datasets from both the bulk and single-cell ESC samples to construct the reference REOs (Additional file 1: Table S3). We further identified the stable REOs in all above ESC samples as reference REO. Finally, the StemSC value of a given sample was calculated as *k*/*n*, where *n* was the number of the referenced REOs contained in this sample and *k* was the number of the gene pairs with same REOs as the reference REOs. Additionally, scripts and codes for the StemSC are available for download (https://github.com/Zhao-Wenyuan/StemSC).

### Construction of the cellular differentiation trajectories

Here, we provided a method to construct the cellular differentiation trajectories by combining StemSC and Monocle 2 [17]. Firstly, to select the highly variable genes for trajectory inference, we removed the genes detected in less than ten cells and selected the top 5000 genes with the largest product of the coefficient of variation square and mean values. Then, the states and branches were detected using the Monocle v2.16.0 R package. Finally, the root of the differentiation process was automatically identified by choosing the state with the highest mean StemSC values.

### Identification of normal cells and the further cell types

Firstly, we downloaded the corresponding normal samples from the GTEx [18] as the reference, unless the original dataset contains peritumoral cells. Next, we used the inferCNV v1.7.1 R package (https://github.com/broadinstitute/inferCNV) to infer the copy number variations (CNV) of all tumor tissue cells by taking these samples as the control. Then, the hierarchical cluster was used to divide these cells into tumor cells with CNV and the normal cells without obvious CNV. We further used SingleR [19] to identify immune cell types for the identified normal cells. In this study, the cell types with less than 50 cells were removed to avoid huge difference in cell numbers.

### Cell–cell communication analysis

The intercellular communication analysis was performed by using CellPhoneDB, a python-based tool [20]. We used CellPhoneDB v2.1.1 python package to analyze the potential interaction networks of the stem-like tumor cells, other common tumor cells and the major types of immune cells.

### Enrichment analysis

The pathway enrichment analyses based on the Kyoto Encyclopedia of Genes and Genomes (KEGG) were conducted and visualized using clusterProfiler v3.16.1 R package [21]. We also used this package to calculate the normalized enrichment score and *p* value for the enrichment of each gene set.

### Statistical analysis

In this study, we used Spearman correlation analysis to evaluate the relationship between StemSC and differentiation time. We also used hypergeometric test to assess the significance of enrichment of the differentially expressed genes in the stem signatures and student T-test to assess the differences of the StemSC values between two types of samples. The Benjamini–Hochberg method was utilized to control the false discovery rate (FDR) in the multiple tests. All statistical analyses were carried out with the R 4.0.2 software package (http://www.r-project.org/).

## Results

### High stability of REOs in single-cell samples

**Fig. 1:**
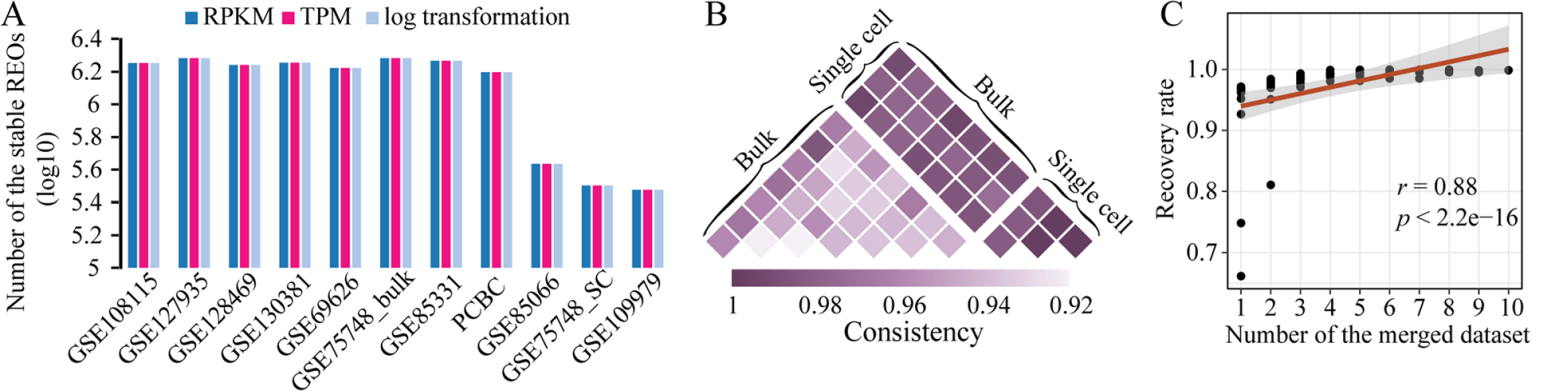
Stability of REOs in both bulk and single-cell ESC samples. **A** The number of stable REOs identified from RPKM, TPM and log transformation data. **B** The consistency of stable REOs among 11 ESC datasets. **C** The correlation between the number of merged datasets for identifying stable REOs and recovery rate of REOs in the remaining datasets

REOs are the features that describe the relative expression orders of gene pairs within a sample. One attribute of their features is that the REOs of RPKM won’t change after within-sample normalization, such as TPM or log transformation. Indeed, the REOs of RPKM showed 100% overlap with those after TPM or log transformation in 11 public human ESC datasets (Fig. 1A, Additional file 1: Table S1). Another attribute is that the REOs stably showing same pattern in the same type of samples, which we called stable REOs (see “Methods” section), could be retained in other independent bulk datasets, even in paraffin-embedded bulk samples with relative low gene expressions [22]. Thus, we inferred that the stable REOs could also be stably retained in the single-cell samples with low gene expressions. Indeed, we found that the stable REOs recognized in each of the 11 single-cell datasets exhibited high consistency among datasets, which was similar for bulk datasets (consistency, 0.99 for single-cell datasets and 0.92–0.96 for bulk datasets, Fig. 1B). Besides, there was also a high consistency of stable REOs between single-cell datasets and bulk datasets (consistency, 0.97–0.99, Fig. 1B). In addition, we found that increasing the number of datasets can improve the stability of REOs in independent datasets (Fig. 1C). The larger the number of the merged datasets, the more stable the stable REOs are.

Considering that the previous REO-based stemness index did not approach the set value 1 in single-cell ESC samples (Additional file 1: Figure S1), it was necessary to add single-cell data to train a signature more suitable for single-cell data. All above results inspired us to add bulk samples to the single-cell samples to build a stemness index that is more suitable for single-cell samples and more robust to batch effects.

### Development and validation of StemSC

**Fig. 2:**
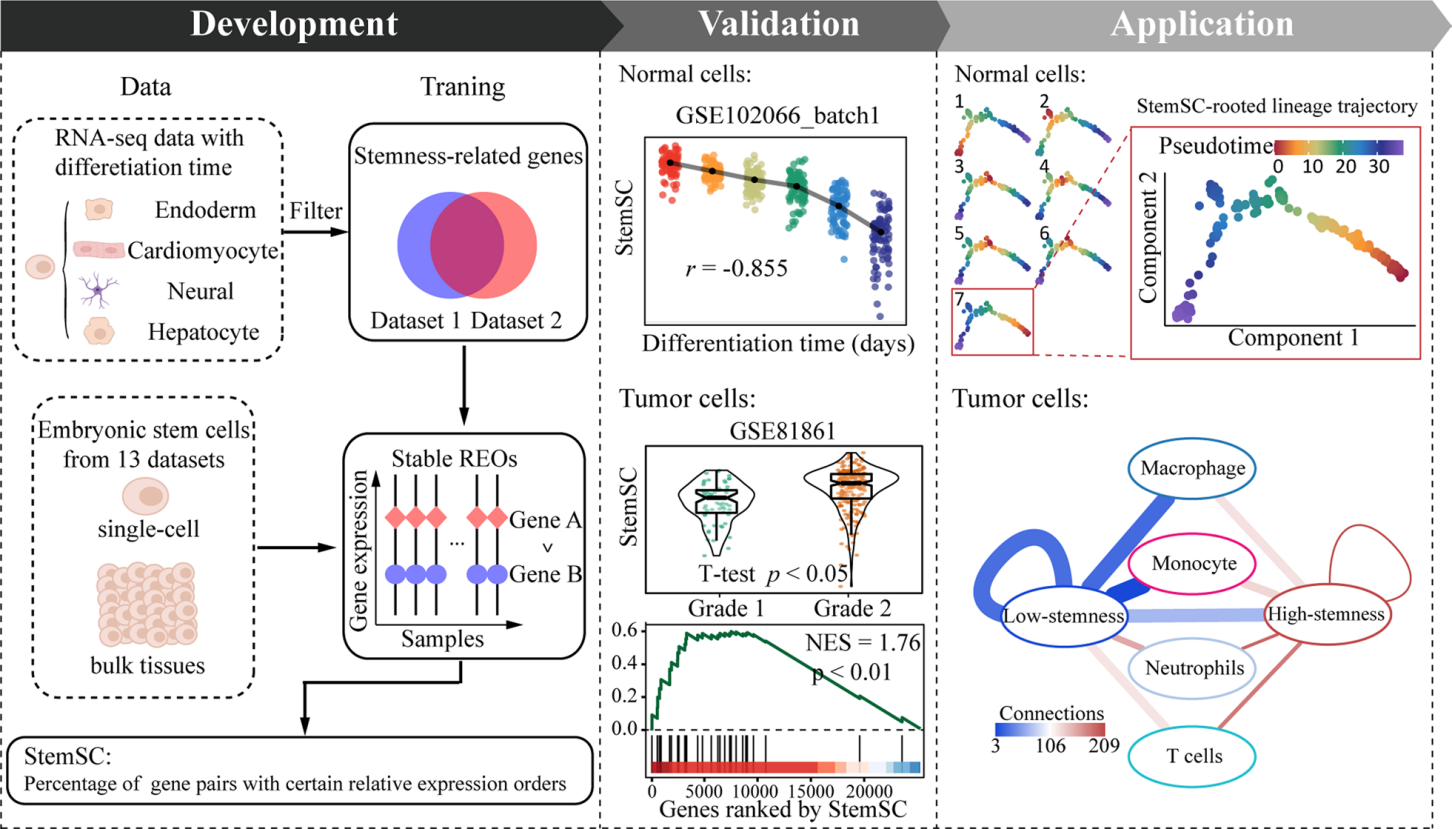
Overall methodology of StemSC

The development procedure of StemSC is shown in Fig. 2. We collected both bulk and single-cell datasets as training sets because of the shortage of single-cell data and the high consistency of REOs between bulk and single-cell datasets (Fig. 1B). Firstly, to reduce redundant REOs, we identified 437 stemness-related genes by choosing the genes which were significantly associated with differentiation time in all five datasets with different differentiation directions (Spearman, FDR < 0.05, Additional file 1: Table S2). Naturally, these genes showed significant enrichment in the pathways related to cell renewal and differentiation, such as cell cycle, Ribosome biogenesis in eukaryotes (Additional file 1: Figure S2). Then, we, respectively, identified 19,937 and 50,827 gene pairs with stable REOs in 92 single-cell and 47 bulk ESC samples from 13 datasets (Additional file 1: Table S3). For the 16,848 shared gene pairs of the above two gene pair lists, 99.9% (16,839/16,848) of them had the same REOs, which showed the high consistency of REOs between single-cell and bulk samples again. Finally, the stemness index values of the given human single cells, StemSC, were calculated by the percentage of gene pairs with the same REOs as ESC samples in 16,839 gene pairs (see “Methods” section).

**Fig. 3:**
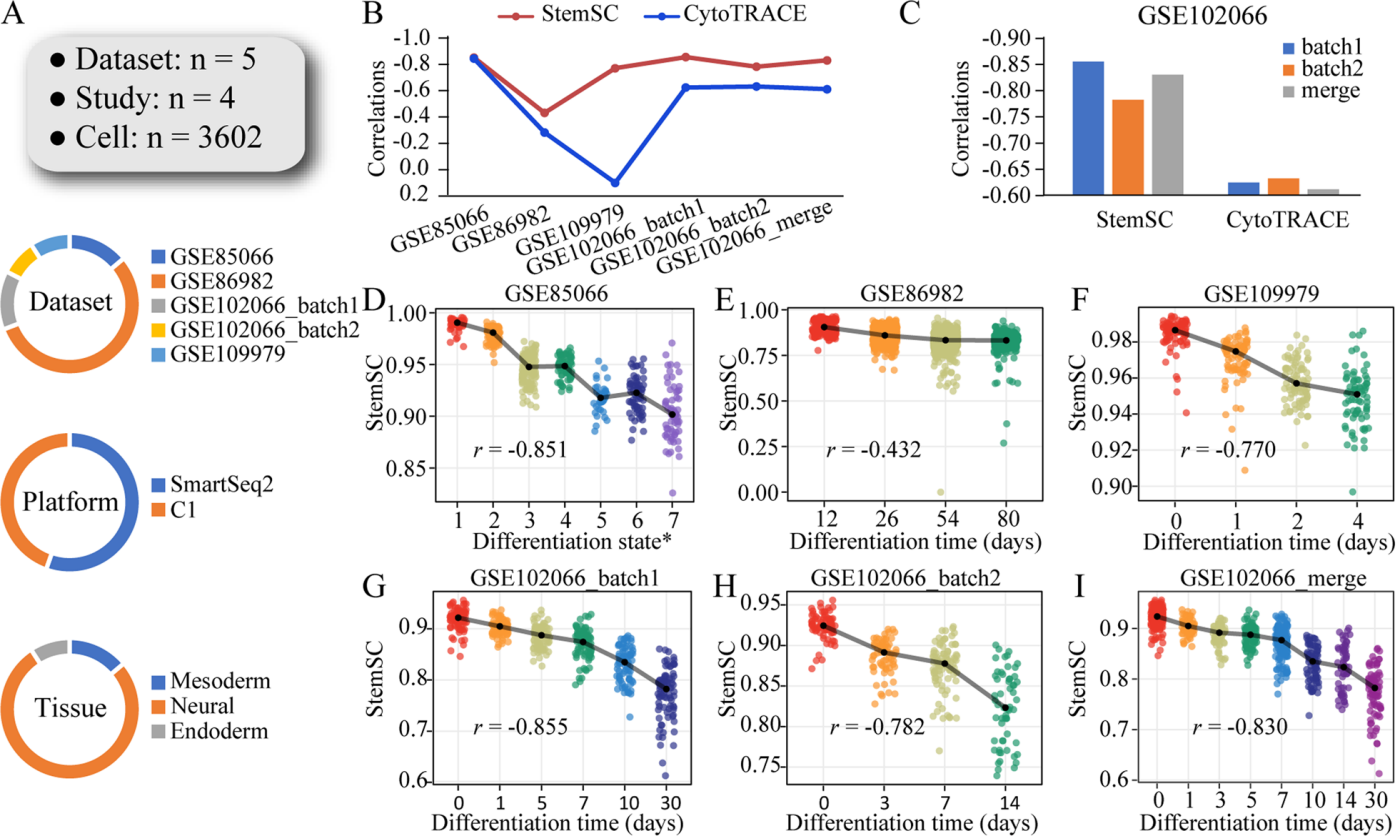
Validation of the StemSC in the single-cell datasets with differentiation time. **A** The general information of validation sets. **B** The correlations between differentiation time and stemness index (StemSC and CytoTRACE) in all validation sets. **C** The changes of correlations between differentiation time and stemness index (StemSC and CytoTRACE) after combining the two batches of GSE102066. **D**–**I** The high correlations differentiation time and StemSC in each validation set. *Differentiation state of dataset GSE85066 was provided in Additional file 1: Table S5

In all five independent validation datasets (Fig. 3A, Additional file 1: Table S4), there were strong negative correlations between StemSC values and differentiation time (Spearman correlation; |*r*|, 0.43–0.85, Fig. 3B, D–H), which was greatly higher than that between CytoTRACE and differentiation time (Spearman correlation; |*r*|, 0.14–0.84, Fig. 3B). Further, the robustness of StemSC to batch effect was showed in three following aspects. Firstly, the median StemSC values of ESCs were all centered around the 1 in two independent validation datasets (median StemSC, 0.990 for GSE85066 and 0.984 for GSE109979). Secondly, after combining the two batches of dataset GSE102066, the correlation between StemSC and differentiation time was higher in the merged data than in one of the single batch data (Fig. 3C, I), but not for CytoTRACE. Thirdly, StemSC showed negative correlations with the differentiation time in all validation sets, but CytoTRACE showed a positive correlation in the dataset GSE109979 (Fig. 3B). In addition, to demonstrate that our method can be applied to the cells with different origins, we deleted the ESC samples from the above two datasets with ESCs (GSE85066 and GSE109979). Results showed that there was still a higher negative correlation between the StemSC values and differentiation time than CytoTRACE (*r*, -0.798 and -0.583 for StemSC; -0.761 and 0.864 for CytoTRACE). This feature could allow the StemSC to be used in the cancer cells with multiple origins.

### The ability of the StemSC to identify stemness-associated genes and construct cellular differentiation trajectories

**Fig. 4:**
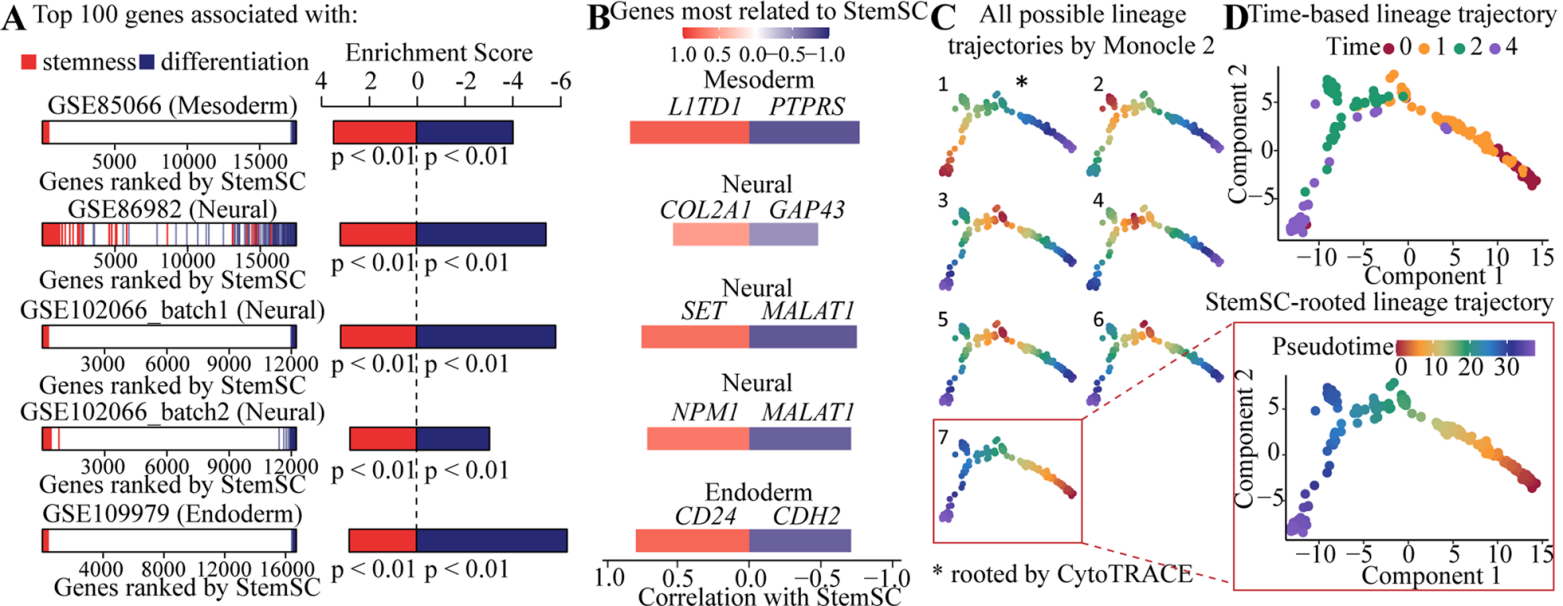
Abilities of StemSC to Identify the stemness-related genes and cellular differentiation trajectories. **A** The enrichment of the top 100 stemness-associated or differentiation-associated genes (the top 100 genes positively or negatively correlated with differentiation time) in the StemSC-ranked gene list. **B** Genes most positively or negatively correlated with StemSC. **C** Construction of lineage trajectory by combining Monocle 2 and StemSC. **D** The time-based lineage trajectory

Given the high correlation between StemSC values and differentiation time, we next explored the ability of the StemSC to identify the stem makers or differentiation factors. Firstly, by ranking all genes according to their correlations with StemSC, we found the enrichment of the stemness-associated genes (the top 100 genes positively correlated with differentiation time) in the positive region and differentiation-associated genes (the top 100 genes negatively correlated with differentiation time) in the negative region in all the validation sets (Fig. 4A). Besides, the majority of the most positive or negative genes showed their role in stemness or differentiation (Fig. 4B, Additional file 1: Table S6). For example, *L1TD1*, the most positively correlated gene with stemness, was reported to be a marker for undifferentiated human ESCs [23]. For another example, *CDH2*, the gene trigging the endodermal germ-layer formation [24], showed the most negative correlation with stemness in the endodermal differentiation samples. All above results showed the potential of StemSC to recognize the tissue-specific and stemness-associated genes.

Cell lineage trajectory can be determined by using transcriptome-based branch detection tools, such as Monocle 2 [17]. However, users need to enter the starting point of the biological processes. For example, when applied to the dataset GSE109979, Monocle 2 constructed seven possible cell trajectories with different roots (Fig. 4C). On the contrary, our method could identify the correct root by choosing the state with the largest average of StemSC values (Fig. 4C), which was similar to time-based lineage trajectory (Fig. 4D). However, CytoTRACE chose the wrong root in this dataset. Similarly, StemSC recognized the correct roots in the four remaining validation sets (Additional file 1: Figure S3). The above results showed a better method to automatically construct cellular differentiation trajectories by combining StemSC and the branch detection tools Monocle 2.

### StemSC can evaluate the stemness of tumor cells

**Fig. 5:**
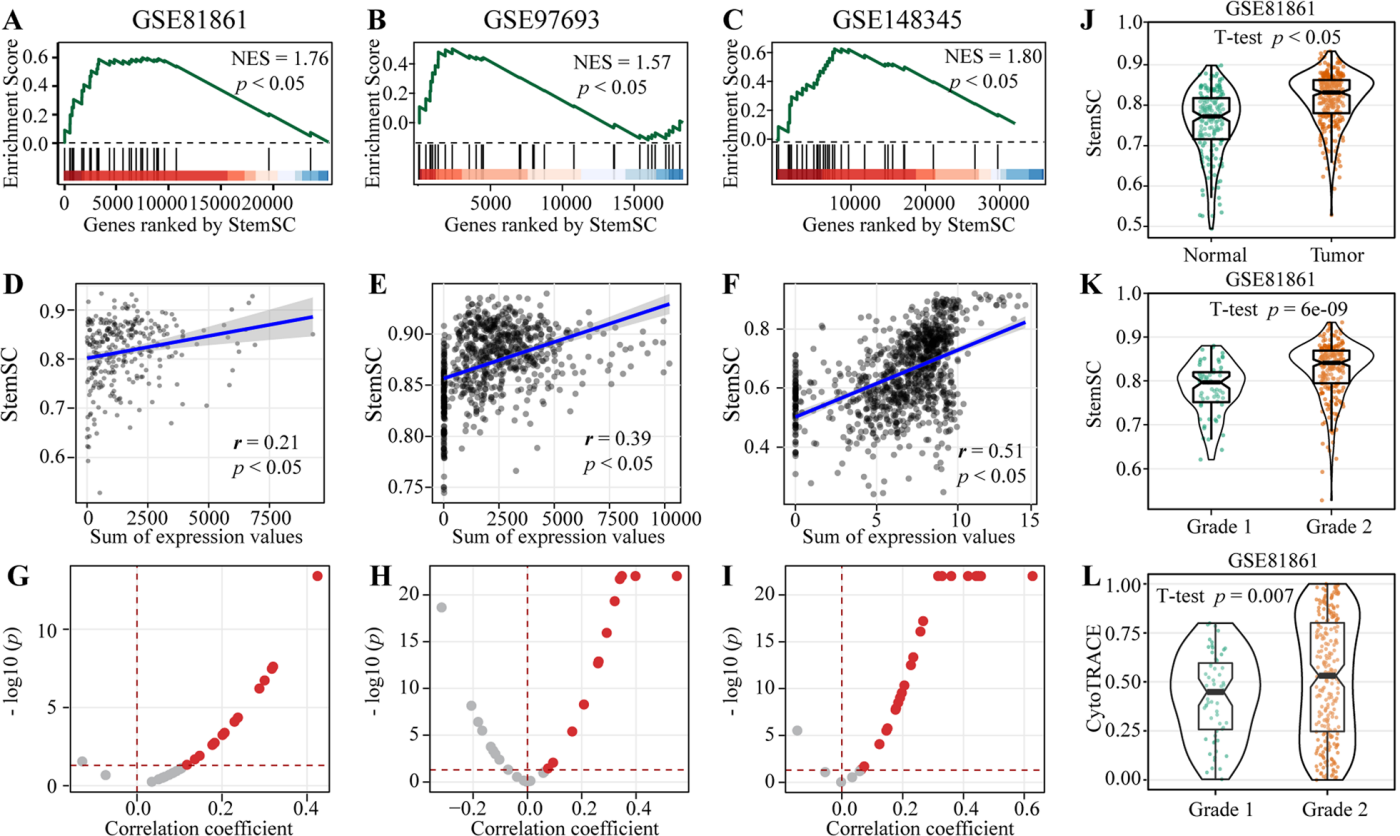
Validation of StemSC in colorectal cancer. **A**–**C** Enrichment of the 30 intestinal stem cell markers in the StemSC-ranked gene list. **D**–**F** The correlation between StemSC and the sum of gene expression values of the 30 intestinal stem cell markers. **G**–**I** The correlations between the StemSC and the gene expression values of 30 intestinal stem cell markers. **J** The significant difference of StemSC between tumor and normal tissue cells. The difference of stemness index between cells with different grades by using StemSC (**K**) and CytoTRACE (**L**)

As reported, Tathiane M. Malta et al. showed the applicability of stemness index in tumor tissues by its positive correlation with oncogenic dedifferentiation [5]. In this study, we used the similar method to study if StemSC can evaluate the stemness of tumor cells at single-cell level, which took the single-cell data of colorectal cancer and glioma (Additional file 1: Table S4) as examples. For three single-cell datasets of colorectal cancer, we found that the 437 stemness-related genes used in StemSC retained their status in colorectal cancer by showing their significant enrichment in the gene list ranked by their correlations with the referential stem values, the sum of expression values of the 30 known intestinal stem markers [25] (Spearman, *p* < 0.05, Additional file 1: Figure S4A). Besides, 120 of the 437 genes were significantly correlated with the referential stem values across all three datasets (Spearman, *p* < 0.05). For example, *HMGA1*, whose expression was significantly correlated with ESC differentiation and the referential stem values of colorectal cancer in our study, is also reported to be highly expressed in human ESCs and poorly differentiated, stem-like cancers [26]. Above results showed the basis of StemSC to evaluate the colorectal cancer stemness. Further, the 30 intestinal stem markers were significantly enriched in the gene list ranked by their correlations with the StemSC values (Fig. 5A–C, Additional file 1: Table S7) and the StemSC values were significantly correlated with the sum of expression values of these 30 markers (Spearman, *p* < 0.05, Fig. 5D–F). What’s more, many of the stem markers showed significantly positive correlations with the StemSC values (Fig. 5G–I). Especially, histological grade reflects the dedifferentiation of cancer tissue. For the dataset GSE81861 with grade information, significantly higher StemSC values were found not only in the 290 tumor tissue cells than the 170 normal tissue cells but also in the 230 low differentiation cells (grade 2) than the 60 high differentiation cells (grade 1) (student T-test, *p* = 6E−9, Fig. 5J, K), which was more significant than the values calculated by CytoTRACE (student T-test, *p* = 0.007, Fig. 5L).

**Fig. 6:**
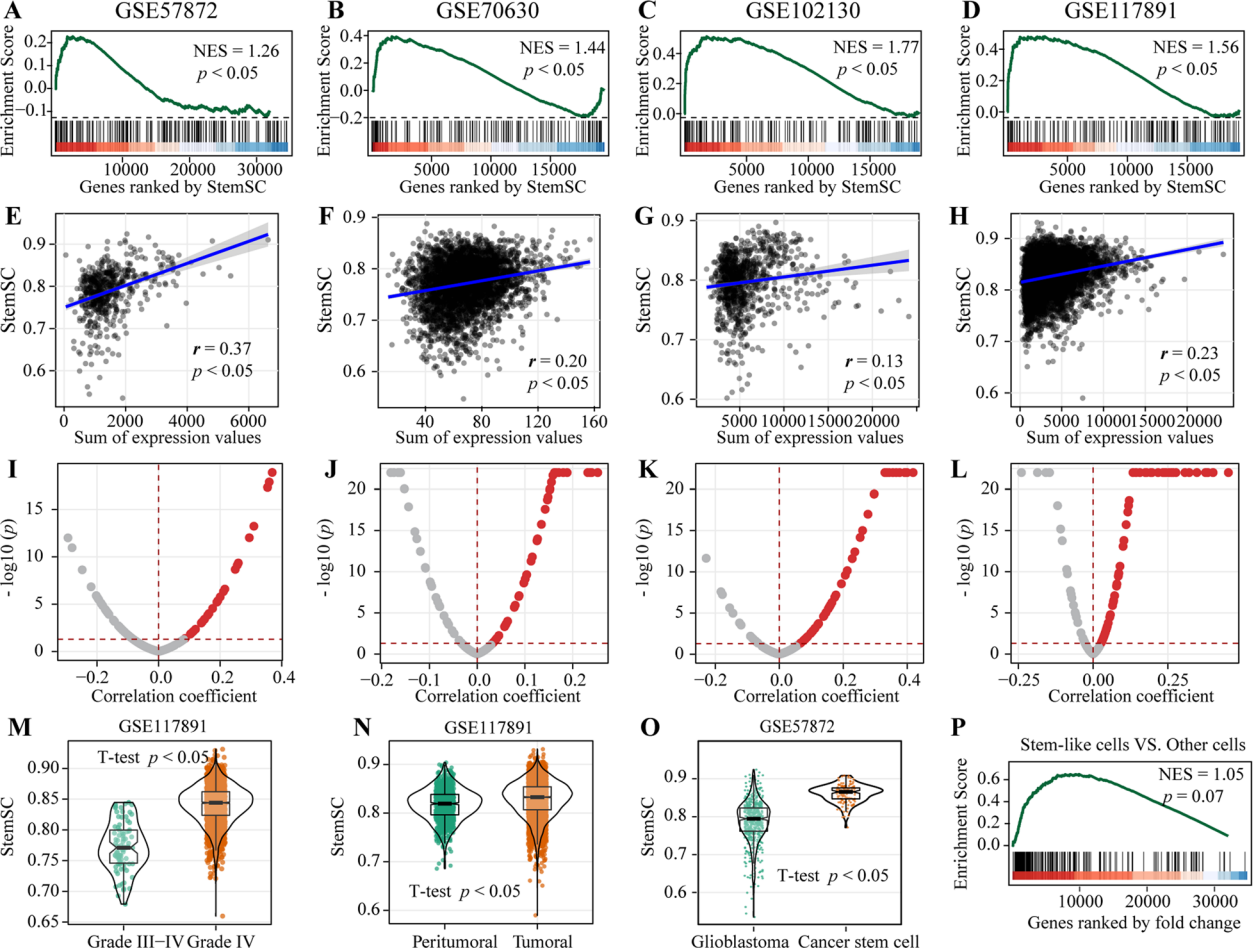
Validation of StemSC in glioma. **A**–**D** Enrichment of the 200 glioma stem markers in the StemSC-ranked gene list. **E**–**H** The correlation between StemSC and the sum of gene expression values of the 200 glioma stem markers. **I**–**L** The correlations between the StemSC and the gene expression values of the 200 glioma stem markers. The significant difference of StemSC between (**M**) different grades (**N**) tumor and normal tissue cells (**O**) CSCs and differentiated cells. **P** Enrichment of the 200 glioma stem markers in the gene sets ranked by the log FCs between stem-like and other common tumor cells

Similarly, for four single-cell datasets of glioma (Additional file 1: Table S4), we firstly derived stem markers from dataset GSE57872 by choosing the top 200 genes with the largest log fold change (FC) values between Glioblastoma stem-like cells and the differentiated cells (edgeR, FDR < 0.05, Additional file 1: Table S7). Then, we found the 437 stemness-related genes used in StemSC also retained their status in glioma by showing their significant enrichment in the gene list ranked by their correlations with the sum of expression values of the 200 stem genes (Spearman, *p* < 0.05, Additional file 1: Figure S4B). For example, *HMGA1* was not only high expressed in human ESC, but also can reduce stemness of glioblastoma stem cells by its silence [26, 27]. Besides, the 200 genes were not only enriched in the StemSC-ranked gene list (Fig. 6A–D) but also correlated with StemSC values (*p* < 0.05, Fig. 6E–L) in both the dataset GSE57872 and the other three independent datasets. Further, for the dataset GSE117891 with grade information, significantly higher StemSC values were not only in the cells with label Grade IV than those with label Grade III–IV but also in the tumoral cells than the peritumoral cells (student T-test, *p* < 0.05, Fig. 6M, N).

### StemSC can recognize the stem-like cells

CSCs are a small subset of tumor cells with the unlimited differentiation ability [28]. And the xenotransplantation assay has become the gold standard assay to define the CSCs in tumor cells [28355176]. However, few study provided such experimentally validated CSCs. In this study, the robust of StemSC to batch effect was showed above, which enables us to leverage the existing experimentally validated CSC samples as the reference to recognize the stem-like cells in other independent datasets. Firstly, we found the StemSC values of the 134 CSCs which were experimentally validated by xenotransplantation were significantly higher than those of the 563 differentiated tumor cells (student T-test, *p* < 0.05, Fig. 6O) in dataset GSE57872, which showed the potential of StemSC to recognize the tumor stem-like cells at the level of stemness. Then, in order to set the proper threshold of StemSC to recognize the stem-like cells, we used the dataset GSE57872, the only dataset with both the CSCs and differentiated tumor cells. And we found the lower limit 0.862 can be used to recognize the stem-like group with the max percentage of CSCs (Additional file 1: Figure S5). Finally, it is necessary to validate that this threshold can divide the samples into two groups with significant difference in stemness. On one hand, we recognized the stem-like cells in the 563 differentiated tumor cells of dataset GSE57872 by the lower limit 0.862. And the stemness markers showed a marginally significant enrichment in the gene sets ranked by the log FCs between stem-like and other cells (Fig. 6P, p = 0.07), which showed the difference of stemness between the two groups at the transcriptome level. On the other hand, similar result was validated in other independent datasets before we used the recognized stem-like cells in the following part.

### The effect of stemness on tumor immune microenvironment

Cancer progression is accompanied by the acquisition of stemness, which greatly affects the immune response of the tumor cells. As reported, the resistance to immune-mediated destruction was shown to be an intrinsic property of CSCs [29]. Similarly, at the bulk tissue level, pervasive negative associations were found between cancer stemness and anticancer immunity [4]. However, at the single-cell level, the effect of stemness on the interaction between tumor cells and tumor microenvironment remains incompletely understood.

**Fig. 7:**
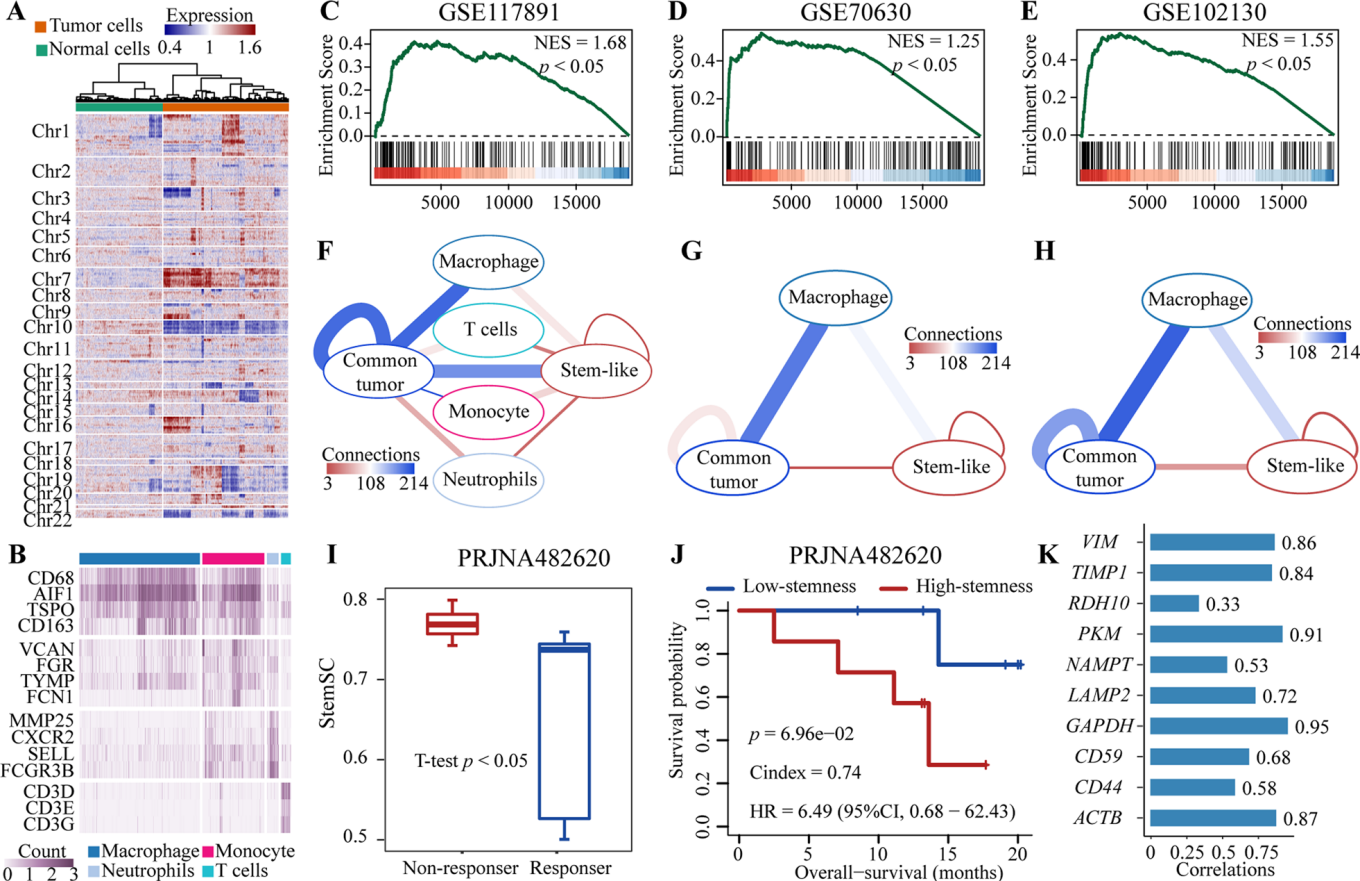
Effect of stemness on tumor immune microenvironment. **A** The hierarchical cluster of the inferred copy number variation in the tumoral tissue cells of dataset GSE117891. **B** The expressions of the corresponding markers for the four types of immune cells in the dataset GSE117891. **C**–**E** The enrichment of the 200 stemness markers in the gene sets ranked by the log FCs between stem-like and other common tumor cells. **F**–**H** Interaction networks among immune cells, stem-like and other common tumor cells. **I** The higher median StemSC values in the non-responders than in the responders. **J** The Kaplan–Meier curves of overall survival in the high- and low-stemness groups. **K** The correlations between StemSC and the expressions of the 10 metastasis-associated genes in glioma

Here, we further divided the cells into different kinds of immune cells, stem-like tumor cells, and other common tumor cells in the above four glioma datasets except dataset GSE57872, which only has tumor cells. For dataset GSE117891, we used inferCNV (see “Methods” section) to infer the copy number variations (CNV) of all the 4623 tumor tissue cells. Further, we divided these cells into 2724 tumor cells and the 1899 normal cells without obvious CNV by using the hierarchical cluster (Fig. 7A, see “Methods” section). For the identified normal cells, we used SingleR [19] to identify the four immune cell types with more than 50 cells, which was further confirmed by the corresponding cell markers (Fig. 7B). For the identified tumor cells, we used the above threshold 0.862 to classify these cells into 664 stem-like and 2060 other common tumor cells, which was confirmed by the enrichment of the 200 stemness markers in the FC-ranked gene sets between the two groups (Fig. 7C). Further, cell–cell communication analysis (see “Methods” section) showed that, the stem-like cells had fewer connections with each other and fewer interactions with the four types of immune cells than other common tumor cells (Fig. 7F). A similar result could be found in the two additional glioma datasets (Fig. 7D, E, G, H), which implied the resistance of high-stemness cells to the immunotherapy. To validate this result, we further collected the bulk RNA-seq samples of 13 immunotherapy-treated patients as the mean values of single-cell samples (dataset PRJNA482620). And we found that the median StemSC values of non-responders were significantly higher than those of responders (student T-test, *p* < 0.05, Fig. 7I). Besides, we used the median values to divide the 13 samples into high- and low-stemness groups. Survival analysis showed that the high-stemness group had marginally significantly worse overall survival than the low-stemness group (HR = 6.49; C-index = 0.74; *p* = 6.96E−2, Fig. 7J), which validated the negative effect of stemness on immunotherapy. Further, due to the lack of other prognostic factors in dataset PRJNA482620, such as metastasis, we used the expression of top 10 genes which are significantly associated with the metastasis of glioma [30] to study the independent prognostic role of StemSC in the immunotherapy-treated patients. Results showed that all 10 genes were significantly positively correlated with StemSC (Pearson, *p* < 0.05, Fig. 7K), which implied that the prognostic role of stemness may be closely dependent on metastasis.

## Discussion

Stemness, which describes the differentiation potential of cells, can be a potential index to construct the cellular differentiation trajectories [1, 3]. Here, we developed a REO-based stemness index called StemSC which could be well applied to single-cell samples across datasets. In addition to the high correlation with differentiation time, StemSC can be combined with Monocle 2 to construct the cellular differentiation trajectories automatically. Besides, the insensitivity of StemSC to batch effects enables it to leverage the existing experimentally validated stem cells to identify the stem-like cells in independent datasets. Finally, we found that the recognized stem-like cells had fewer interactions with the immune cells than the common tumor cells. And the immunotherapy-treated patients with higher stemness had worse overall survival than those with lower stemness.

REO is the feature that transforms the continuous expression values into discrete values. Thus, using REOs instead of the absolute values of gene expression can effectively avoid overfitting and outliers in the training process. Besides, the larger the numbers of training datasets, the more stable the REO-based signatures are. However, due to the insufficiency of the datasets with differentiation time information and the single-cell samples of ESCs, we chose the limited datasets for training StemSC. When testing the application of StemSC, we found that StemSC can only recognize the stem-like cells instead of CSCs, which may result from the mixed stemness between CSCs and other common tumor cells (Fig. 6O). If the datasets of the experimentally validated CSCs are sufficiency enough, it is possible to build a REO-based classifier to identify the CSCs individually. Besides, the tumor datasets we collected had limited clinical features. We extracted the metastasis information from tumor stage by taking the stage IV samples (distal metastasis) as the metastatic tumor. Only one dataset with both the metastatic and non-metastatic primary tumor was collected for both colorectal cancer and glioma. And we found that the relationships between tumor stemness and metastasis were different for the two tumor types (Additional file 1: Figure S6). Such result was similar to the finding that, although stemness was higher in metastatic samples than primary tumors in most cases (6 of the 9 tumor types), there were still some tumor types with the contradictory relationships [5]. On the other hand, different relationships may result from the limited samples and datasets. Thus, more comprehensive datasets are needed to make the conclusion. Besides, in this study, we only showed the applicability of StemSC in ESC differentiation and tumor cells, but its applicability in other samples is still unknown. Thus, it should be verified before StemSC is used in other samples.

In the future work, it is hopeful to recognize the CSCs individually when there are enough single-cell samples of CSCs. Besides, the development of StemSC can help other researchers to study the cell differentiation trajectories. When applying StemSC to tumor tissue cells, we found that the stem-like tumor cells had fewer connections with immune cells. The deeper investigations of this phenomenon may reveal new mechanisms of immune cell regulation and provide a new direction for immunotherapy.

## Conclusion

We constructed a REO-based stemness index for the single-cell samples, StemSC, which showed high correlations with the differentiation time of embryonic stem cells and high correlations with tumor dedifferentiation. In addition to its ability to construct cellular trajectories, StemSC could also be used to recognized the stem-like tumor cells across datasets and reveal that the recognized stem-like cells had fewer connections with anti-tumor immune cells.

## Supplementary Information

**Additional file 1:** Supplementary data for StemSC.

## Data Availability

The datasets supporting the conclusions of this article are available in the in the Gene Expression Omnibus (GEO, https://www.ncbi.nlm.nih.gov/geo/) and Progenitor Cell Biology Consortium (PCBC, https://www.synapse.org/#!Synapse:syn1773109/wiki/54962).

## Abbreviations

StemSC: Stemness index for single-cell samples
CSC: Cancer stem cell
REOs: Relative expression orderings
ESC: Embryonic stem cell
CNV: Copy number variations
FDR: False discovery rate
KEGG: Kyoto Encyclopedia of Genes and Genomes

## References

[1] Wu J, Belmonte JCI (2016). Stem cells: a renaissance in human biology research. Cell.

[2] Thorgeirsson SS (2016). Stemness and reprogramming in liver cancer. Hepatology.

[3] Gulati GS, Sikandar SS, Wesche DJ, Manjunath A, Bharadwaj A, Berger MJ, Ilagan F, Kuo AH, Hsieh RW, Cai S, Zabala M, Scheeren FA, Lobo NA, Qian D, Yu FB, Dirbas FM, Clarke MF, Newman AM (2020). Single-cell transcriptional diversity is a hallmark of developmental potential. Science.

[4] Miranda A, Hamilton PT, Zhang AW, Pattnaik S, Becht E, Mezheyeuski A, Bruun J, Micke P, de Reynies A, Nelson BH (2019). Cancer stemness, intratumoral heterogeneity, and immune response across cancers. Proc Natl Acad Sci USA.

[5] Malta TM, Sokolov A, Gentles AJ, Burzykowski T, Poisson L, Weinstein JN, Kaminska B, Huelsken J, Omberg L, Gevaert O, Colaprico A, Czerwinska P, Mazurek S, Mishra L, Heyn H, Krasnitz A, Godwin AK, Lazar AJ, Cancer Genome Atlas Research N, Stuart JM, Hoadley KA, Laird PW, Noushmehr H, Wiznerowicz M. Machine learning identifies stemness features associated with oncogenic dedifferentiation. Cell. 2018;173:338-354 e315. 10.1016/j.cell.2018.03.034 PMC5902191 29625051

[6] Shimokawa M, Ohta Y, Nishikori S, Matano M, Takano A, Fujii M, Date S, Sugimoto S, Kanai T, Sato T (2017). Visualization and targeting of LGR5(+) human colon cancer stem cells. Nature.

[7] Zheng H, Song K, Fu Y, You T, Yang J, Guo W, Wang K, Jin L, Gu Y, Qi L, Zhao W (2020). An absolute human stemness index associated with oncogenic dedifferentiation. Brief Bioinform.

[8] Ao L, Guo Y, Song X, Guan Q, Zheng W, Zhang J, Huang H, Zou Y, Guo Z, Wang X (2017). Evaluating hepatocellular carcinoma cell lines for tumour samples using within-sample relative expression orderings of genes. Liver Int.

[9] Chen R, He J, Wang Y, Guo Y, Zhang J, Peng L, Wang D, Lin Q, Zhang J, Guo Z, Li L (2019). Qualitative transcriptional signatures for evaluating the maturity degree of pluripotent stem cell-derived cardiomyocytes. Stem Cell Res Ther.

[10] Zheng H, Song K, Fu Y, You T, Yang J, Guo W, Wang K, Jin L, Gu Y, Qi L, Zhao W, Guo Z (2020). A qualitative transcriptional signature for determining the grade of colorectal adenocarcinoma. Cancer Gene Ther.

[11] Barrett T, Wilhite SE, Ledoux P, Evangelista C, Kim IF, Tomashevsky M, Marshall KA, Phillippy KH, Sherman PM, Holko M, Yefanov A, Lee H, Zhang N, Robertson CL, Serova N, Davis S, Soboleva A (2013). NCBI GEO: archive for functional genomics data sets–update. Nucleic Acids Res.

[12] Daily K, Ho Sui SJ, Schriml LM, Dexheimer PJ, Salomonis N, Schroll R, Bush S, Keddache M, Mayhew C, Lotia S, Perumal TM, Dang K, Pantano L, Pico AR, Grassman E, Nordling D, Hide W, Hatzopoulos AK, Malik P, Cancelas JA, Lutzko C, Aronow BJ, Omberg L (2017). Molecular, phenotypic, and sample-associated data to describe pluripotent stem cell lines and derivatives. Sci Data.

[13] Patel AP, Tirosh I, Trombetta JJ, Shalek AK, Gillespie SM, Wakimoto H, Cahill DP, Nahed BV, Curry WT, Martuza RL, Louis DN, Rozenblatt-Rosen O, Suva ML, Regev A, Bernstein BE (2014). Single-cell RNA-seq highlights intratumoral heterogeneity in primary glioblastoma. Science.

[14] Harrow J, Frankish A, Gonzalez JM, Tapanari E, Diekhans M, Kokocinski F, Aken BL, Barrell D, Zadissa A, Searle S, Barnes I, Bignell A, Boychenko V, Hunt T, Kay M, Mukherjee G, Rajan J, Despacio-Reyes G, Saunders G, Steward C, Harte R, Lin M, Howald C, Tanzer A, Derrien T, Chrast J, Walters N, Balasubramanian S, Pei B, Tress M, Rodriguez JM, Ezkurdia I, van Baren J, Brent M, Haussler D, Kellis M, Valencia A, Reymond A, Gerstein M, Guigo R, Hubbard TJ (2012). GENCODE: the reference human genome annotation for The ENCODE Project. Genome Res.

[15] Kim D, Paggi JM, Park C, Bennett C, Salzberg SL (2019). Graph-based genome alignment and genotyping with HISAT2 and HISAT-genotype. Nat Biotechnol.

[16] Liao Y, Smyth GK, Shi W (2014). featureCounts: an efficient general purpose program for assigning sequence reads to genomic features. Bioinformatics.

[17] Qiu X, Mao Q, Tang Y, Wang L, Chawla R, Pliner HA, Trapnell C (2017). Reversed graph embedding resolves complex single-cell trajectories. Nat Methods.

[18] Consortium GT, Laboratory DA, Coordinating Center -Analysis Working G, Statistical Methods groups-Analysis Working G, Enhancing Gg, Fund NIHC, Nih/Nci, Nih/Nhgri, Nih/Nimh, Nih/Nida, Biospecimen Collection Source Site N, Biospecimen Collection Source Site R, Biospecimen Core Resource V, Brain Bank Repository-University of Miami Brain Endowment B, Leidos Biomedical-Project M, Study E, Genome Browser Data I, Visualization EBI, Genome Browser Data I, Visualization-Ucsc Genomics Institute UoCSC, Lead a, Laboratory DA, Coordinating C, management NIHp, Biospecimen c, Pathology, QTLmwg e, Battle A, Brown CD, Engelhardt BE, Montgomery SB. Genetic effects on gene expression across human tissues. Nature. 2017;550:204–13.

[19] Aran D, Looney AP, Liu L, Wu E, Fong V, Hsu A, Chak S, Naikawadi RP, Wolters PJ, Abate AR, Butte AJ, Bhattacharya M (2019). Reference-based analysis of lung single-cell sequencing reveals a transitional profibrotic macrophage. Nat Immunol.

[20] Efremova M, Vento-Tormo M, Teichmann SA, Vento-Tormo R (2020). Cell PhoneDB: inferring cell-cell communication from combined expression of multi-subunit ligand-receptor complexes. Nat Protoc.

[21] Yu G, Wang LG, Han Y, He QY (2012). clusterProfiler: an R package for comparing biological themes among gene clusters. OMICS.

[22] Chen R, Guan Q, Cheng J, He J, Liu H, Cai H, Hong G, Zhang J, Li N, Ao L, Guo Z (2017). Robust transcriptional tumor signatures applicable to both formalin-fixed paraffin-embedded and fresh-frozen samples. Oncotarget.

[23] Wong RC, Ibrahim A, Fong H, Thompson N, Lock LF, Donovan PJ (2011). L1TD1 is a marker for undifferentiated human embryonic stem cells. PLoS ONE.

[24] Giger FA, David NB (2017). Endodermal germ-layer formation through active actin-driven migration triggered by N-cadherin. Proc Natl Acad Sci USA.

[25] Dalerba P, Kalisky T, Sahoo D, Rajendran PS, Rothenberg ME, Leyrat AA, Sim S, Okamoto J, Johnston DM, Qian D, Zabala M, Bueno J, Neff NF, Wang J, Shelton AA, Visser B, Hisamori S, Shimono Y, van de Wetering M, Clevers H, Clarke MF, Quake SR (2011). Single-cell dissection of transcriptional heterogeneity in human colon tumors. Nat Biotechnol.

[26] Ben-Porath I, Thomson MW, Carey VJ, Ge R, Bell GW, Regev A, Weinberg RA (2008). An embryonic stem cell-like gene expression signature in poorly differentiated aggressive human tumors. Nat Genet.

[27] Colamaio M, Tosti N, Puca F, Mari A, Gattordo R, Kuzay Y, Federico A, Pepe A, Sarnataro D, Ragozzino E, Raia M, Hirata H, Gemei M, Mimori K, Del Vecchio L, Battista S, Fusco A (2016). HMGA1 silencing reduces stemness and temozolomide resistance in glioblastoma stem cells. Expert Opin Ther Targets.

[28] Huo X, Han S, Wu G, Latchoumanin O, Zhou G, Hebbard L, George J, Qiao L (2017). Dysregulated long noncoding RNAs (lncRNAs) in hepatocellular carcinoma: implications for tumorigenesis, disease progression, and liver cancer stem cells. Mol Cancer.

[29] Bruttel VS, Wischhusen J (2014). Cancer stem cell immunology: key to understanding tumorigenesis and tumor immune escape?. Front Immunol.

[30] Yuan H, Yan M, Zhang G, Liu W, Deng C, Liao G, Xu L, Luo T, Yan H, Long Z, Shi A, Zhao T, Xiao Y, Li X (2019). CancerSEA: a cancer single-cell state atlas. Nucleic Acids Res.

