# Supplementary material for: StemSC: a cross-dataset human stemness index for single-cell samples
Source: Stem Cell Res Ther. 2022 Mar 21;13:115. doi: 10.1186/s13287-022-02803-5 (PMC8935746; doi:10.1186/s13287-022-02803-5)
Supplement: Supplementary file 1 — Additional file 1: Supplementary data for StemSC. [file 13287_2022_2803_MOESM1_ESM.docx]

**SUPPLEMENTARY DATA**

**StemSC: A cross-dataset human stemness index for single-cell samples**

Hailong Zheng, Jiajing Xie, Kai Song, Jing Yang, Huiting Xiao, Jiashuai Zhang, Keru Li, Rongqiang Yuan, Yuting Zhao, Yunyan Gu and Wenyuan Zhao

Supplementary data include 6 Supplementary figures, and 7 Supplementary tables.

**Supplementary Figure S1.** The previous REO-based stemness index in the single-cell samples of embryonic stem cells.

**Supplementary Figure S2.** The enrichment pathways of the stemness-related genes.

**Supplementary Figure S3.** The automatically constructed cellular differentiation trajectories in the four remaining validation sets.

**Supplementary Figure S4.** Enrichment of the 437 stemness-related genes.

**Supplementary Figure S5.** The relationship between lower limit of StemSC values and the percentage of CSCs in dataset GSE57872.

**Supplementary Figure S6.** The difference of stemness between the metastatic and non-metastatic primary tumor cells for colorectal cancer and glioma.

**Supplementary Table S1.** The 11 public datasets of embryonic stem cells.

**Supplementary Table S2.** The 5 public datasets with differentiation time.

**Supplementary Table S3.** The 13 training datasets with embryonic stem cells.

**Supplementary Table S4.** The validation sets for both normal and tumor cells.

**Supplementary Table S5.** Differentiation state of dataset GSE85066.

**Supplementary Table S6.** The genes most positively or negatively related to the StemSC.

**Supplementary Table S7.** The stemness markers for colorectal cancer and glioma.

**Supplementary Figures**


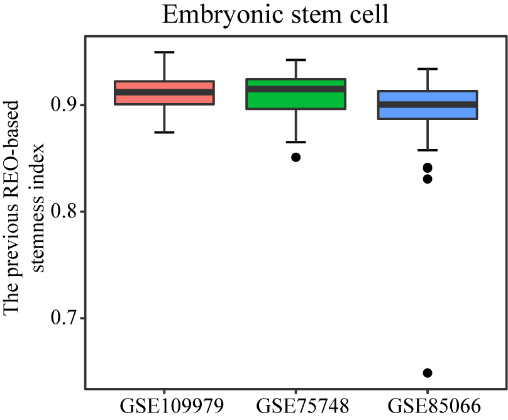


**Supplementary Figure S1.** The previous REO-based stemness index in the single-cell samples of embryonic stem cells.


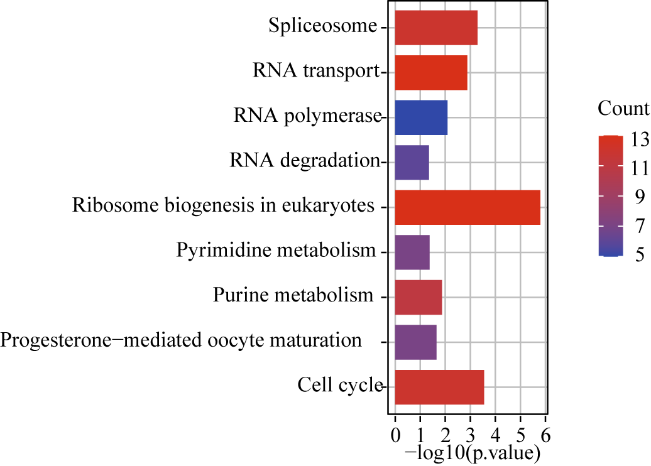


**Supplementary Figure S2.** The enrichment pathways of the stemness-related genes.


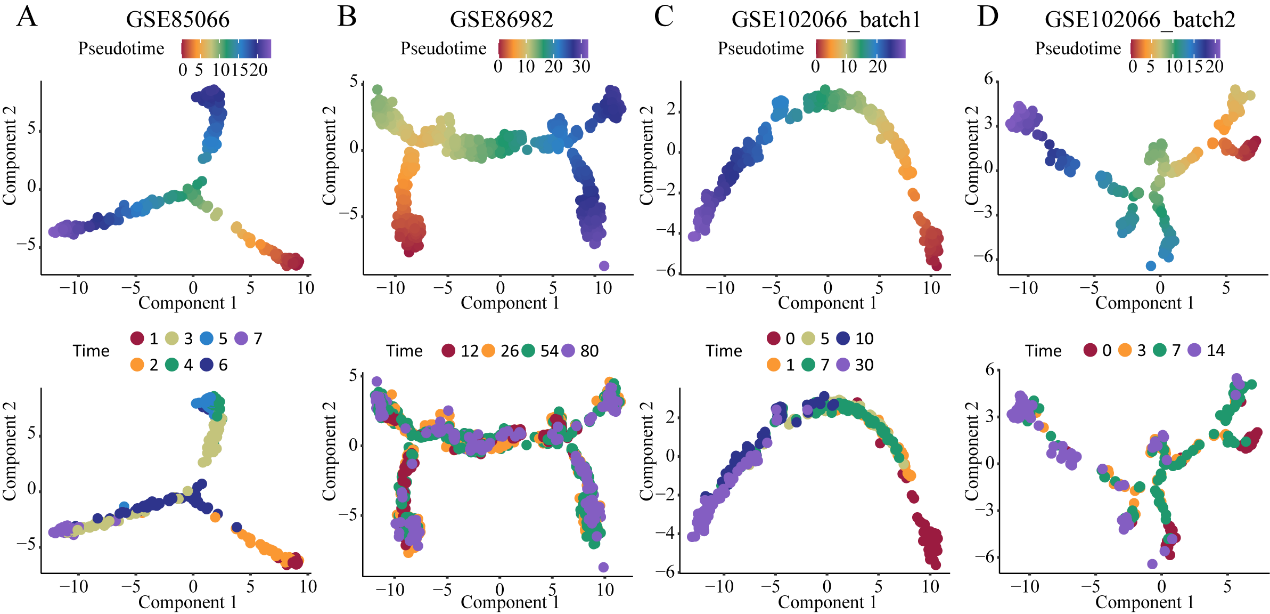


**Supplementary Figure S3.** The automatically constructed cellular differentiation trajectories in the four remaining validation sets.


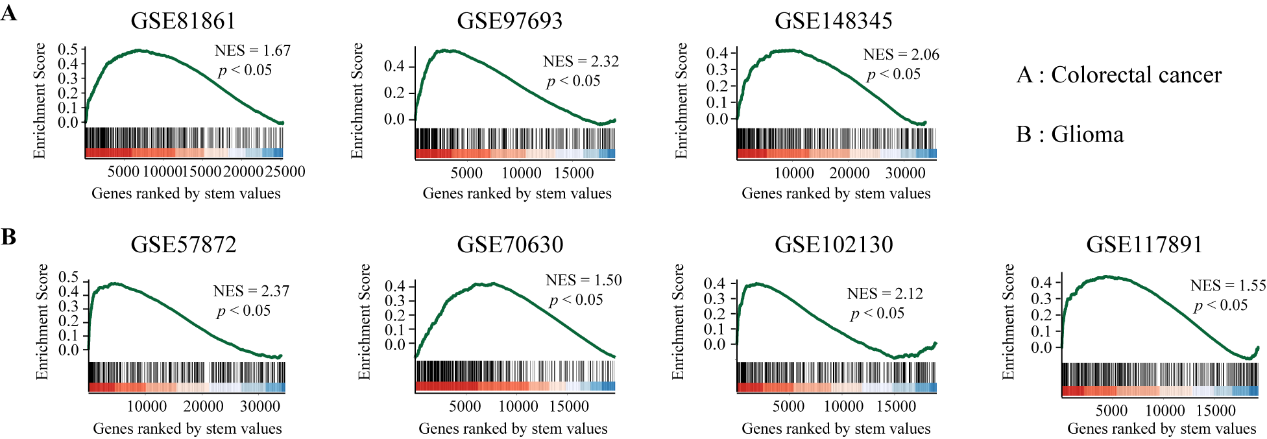


**Supplementary Figure S4.** Enrichment of the 437 stemness-related genes in the gene list ranked by their correlations with the referential stem values of (A) colorectal cancer and (B) glioma


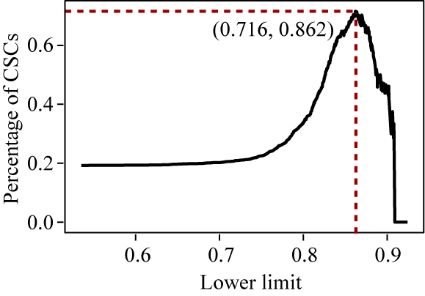


**Supplementary Figure S5.** The relationship between lower limit of StemSC values and the percentage of CSCs in dataset GSE57872.

**
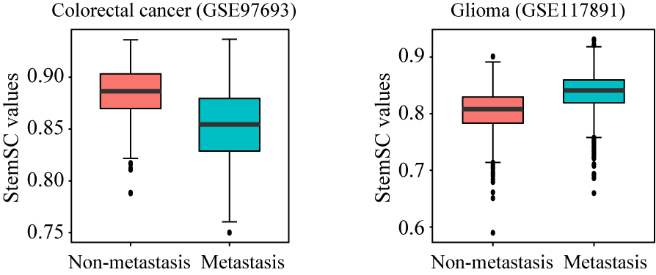
**

**Supplementary Figure S6.** The difference of stemness between the metastatic and non-metastatic primary tumor cells for colorectal cancer and glioma.

**Supplementary Tables**

**Supplementary Table S1.** The 11 public datasets of embryonic stem cells.

| Tissue type | Accession number | Data type | Number of genes | Number of samples |
| --- | --- | --- | --- | --- |
| Bulk | GSE108115 | Count | 28935 | 5 |
|  | GSE127935 | TPM/FPKM | 58051 | 3 |
|  | GSE128469 | TPM | 19084 | 3 |
|  | GSE130381 | Count | 43412 | 4 |
|  | GSE69626 | RPKM | 19929 | 6 |
|  | GSE75748 | Count | 19097 | 3 |
|  | GSE85331 | FPKM | 24669 | 4 |
|  | PCBC | RPKM | 35484 | 14 |
| Single cell | GSE85066 | TPM | 25064 | 51 |
|  | GSE75748 | Count | 19097 | 92 |
|  | GSE109979 | RPKM | 19685 | 84 |

**Supplementary Table S2.** The 5 public datasets with differentiation time.

| Tissue type | Accession number | Differentiation type | Number of samples |
| --- | --- | --- | --- |
| Bulk | GSE90053 | Neural differentiation | 26 |
|  | GSE70741 | Hepatocyte differentiation | 10 |
|  | GSE76523 | Cardiomyocyte differentiation | 11 |
|  | GSE75748 | Endoderm differentiation | 18 |
| Single cell | GSE75748 | Endoderm differentiation | 758 |

**Supplementary Table S3.** The 13 training datasets with embryonic stem cells.

| Tissue type | Accession number | Data type | Number of genes | Number of samples |
| --- | --- | --- | --- | --- |
| Bulk | PCBC | RPKM | 35484 | 14 |
|  | GSE85331 | FPKM | 24669 | 4 |
|  | GSE110504 | TPM | 58219 | 2 |
|  | GSE69626 | RPKM | 19929 | 6 |
|  | GSE108115 | Count | 28935 | 5 |
|  | GSE128469 | TPM | 19084 | 3 |
|  | GSE127935 | TPM/FPKM | 58051 | 3 |
|  | GSE130381 | Count | 43412 | 4 |
|  | GSE90053 | TPM | 19097 | 1 |
|  | GSE70741 | Count | 12125 | 1 |
|  | GSE76523 | FPKM | 41495 | 1 |
|  | GSE75748 | Count | 19097 | 3 |
| Single cell | GSE75748 | Count | 19097 | 92 |

**Supplementary** **Table S4.** The validation sets for both normal and tumor cells.

| Data type | Tissue type | Accession number | Platform | Number of samples* |
| --- | --- | --- | --- | --- |
| Normal | Mesoderm | GSE85066 | C1 | 498 |
|  | Neural | GSE86982 | SmartSeq2 | 1994 |
|  |  | GSE102066_batch1 | C1 | 483 |
|  |  | GSE102066_batch2 | C1 | 298 |
|  | Endoderm | GSE109979 | C1 | 329 |
| Tumor | Colorectal cancer | GSE81861 | C1 | 460 |
|  |  | GSE97693 | C1/scTrio-seq2 | 1096 |
|  |  | GSE148345 | SmartSeq2 | 1426 |
|  | Glioma | GSE57872 | SmartSeq | 563 |
|  |  | GSE102130 | SmartSeq2 | 3057 |
|  |  | GSE70630 | SmartSeq2 | 4347 |
|  |  | GSE117891 | C1 | 4653 |

* We used the cells with more than 2000 detected genes

**Supplementary** **Table S5.** Differentiation state of dataset GSE85066.

| Cell type | Differentiation state |
| --- | --- |
| H7hESC | 1 |
| Anteriorprimitivestreak (APS) | 2 |
| Middleprimitivestreak (MPS) | 2 |
| Lateralmesoderm (LatM) | 3 |
| Paraxialmesoderm (PXM) | 3 |
| Somitomere | 4 |
| Earlysomite | 5 |
| Dermomyotome (DM) | 6 |
| Sclerotome | 7 |

**Supplementary** **Table S6.** The genes most positively or negatively related to the StemSC.

| Tissue type | Accession number | Gene symbol | Correlation coefficient | PMID |
| --- | --- | --- | --- | --- |
| Mesoderm | GSE85066 | *PTPRS* | -0.77 | - |
|  |  | *L1TD1* | 0.83 | 21559406 |
| Neural | GSE86982 | *GAP43* | -0.48 | 32633468 |
|  |  | *COL2A1* | 0.53 | - |
|  | GSE102066_batch1 | *MALAT1* | -0.75 | - |
|  |  | *SET* | 0.75 | 33296674 |
|  | GSE102066_batch2 | *MALAT1* | -0.71 | - |
|  |  | *NPM1* | 0.71 | 16870143 |
| Endoderm | GSE109979 | *CDH2* | -0.71 | 28874564 |
|  |  | *CD24* | 0.79 | 26076835 |

**Supplementary** **Table S7.** The stemness markers for colorectal cancer and glioma.

| Cancer types | Markers |
| --- | --- |
| Colorectal cancer | LGR5, AQP1, EZH2, GPSM2, METTL3, OLFM4, PTPRO, UGT8, VEGFA, AXIN2, BMI1, CDK6, CFTR, HES1, MYC, NOTCH1, DNMT3A, KIF12, MLLT10, RGMB, RNF43, EGFR, LRIG1, CXCL2, CDCA7, FERMT1, TSPAN6, STMN1, DPP4, ASCL2 |
| Glioma | PDXDC2P, MAGEC2, XIST, HOMEZ, SP110, TMSB15A, MAN2B2, MAGEA12, NMNAT2, CSAG1, TOM1L2, RAB44, MAGEA3, GALC, PMAIP1, SIGLEC10, MAGEA6, CRYBB2P1, MYCN, MAGEB2, MIA, MYO5A, CPM, MAGEC1, GACAT3, SIX6, EBLN1, FAM110D, RPL39L, MAGEB1, PDGFRA, BMS1P22, EIF3I, NQO1, CHRDL1, GSX2, MYCNOS, ERFE, SOX10, ASB9, PGBD4P6, MYCNUT, STC1, PLA2G3, MCM10, PDK1P2, SLFN11, LINC01804, MTCYBP38, CNIH3-AS2, MRO, LNCOC1, SLC35D1, PTER, FAM170B, ANKRD26P1, GNG11, FGFBP3, KLRC2, CYTL1, TFPI2, RNU5E-7P, CCR7, OR13F1, PRAME, SFRP2, PDP1, GALNT14, CDK6-AS1, FDFT1, FMOD, RPLP1P5, GABRA5, PIR, INHBE, L1CAM, PABPC5, LAMP5, NR0B1, ZNF280A, MAGEB6, SNRPEP4, MC4R, SLCO4A1, DLK1, SH3KBP1, TCF21, SEMA3A, SLC25A21-AS1, FAM166C, ACAT2, SNRPEP2, BLACAT1, NDN, RPL21P44, ARHGAP19-SLIT1, LINC02175, SQLE, FABP3, MT-TM, SEMA3E, CHIC2, CAMKV, TUBB6, H1-9P, GINS2, SNRPE, CDCA7, TYRO3P, UBAC2-AS1, MAGEB10, CGAS, MFN1P1, CALCOCO2, TMEM97, RGS10, IDI1, CENPN, C10orf120, TEX13D, KCNA7, FAM47B, MKRN9P, UCP2, OR1Q1, KLF11, CBX2, HAPLN1, FLRT3, FUOM, NXPH2, MROH6, HEY2, COL2A1, SLC1A5, H2ACP1, PDLIM1, SLCO4A1-AS1, KRTAP27-1, STK26, HMGB3P30, CENPW, RN7SL104P, ITIH6, EPHA3, TRAV4, HSPD1P2, RAD23BP1, LGI2, RNA5SP525, ERBB3, PGAP4, RPS28P7, BIRC5, CD40, LGR5, APOBEC3B, NR2F2, ATP9A, S100A11, PBK, SLC4A11, RAB3B, DOK4, RAET1K, SLC29A1, CKS2, VIM2P, NCAPD2, GLB1L2, BAMBI, KLRC4-KLRK1, UBE2T, CKS1BP3, MAD2L1P1, CBX3P9, SPIN2P1, DTL, HRH2, CYSRT1, DHH, LPP-AS2, TNP2, PCLAF, RPS4XP11, SETD6P1, XKR5, ESCO2, CENPM, ACTRT2, OR7E148P, LINC01126, MASTL, HMGCR, DDX18P3, MDM2, SPESP1, TXNP4, LINC02283, LINC00703 |
